# Successful endovascular treatment for retroflexed anterior tibial artery origin in chronic limb-threatening ischemia

**DOI:** 10.1016/j.radcr.2026.08.057

**Published:** 2026-09-07

**Authors:** Tetsuya Nomura, Shogo Kaketaka, Tokuaki Bando, Tomonao Mizutani, Mai Imanaka, Jun Munakata, Hiroshi Kubota, Naotoshi Wada, Natsuya Keira, Tetsuya Tatsumi

**Affiliations:** Department of Cardiovascular Medicine, Kyoto Chubu Medical Center, 25, Yagi-Ueno, Yagi-cho, Nantan City, Kyoto 629-0197, Japan

**Keywords:** Endovascular treatment, Anterior tibial artery, Retroflexed origin, Retrograde approach

## Abstract

The success rate of endovascular treatment (EVT) for infrapopliteal lesions has increased significantly; however, EVT can be particularly challenging when the anterior tibial artery (ATA) originates in a retroflexed configuration. An 86-year-old man presented with rest pain in the right lower extremity (Rutherford category 4). Angiography revealed severe proximal ATA stenosis. Multiple attempts at antegrade wiring failed because of difficulty in engaging the ATA origin. Therefore, a retrograde approach via the dorsalis pedis artery was established. Retrograde angiography demonstrated that the ATA originated from a retroflexed branch of the popliteal artery (POPA). Initially, the retrograde guidewire advanced in a subintimal course toward the POPA, but eventually, the guidewire successfully passed along the course of the blood vessel. However, proximal reversal of direction was difficult using guidewire manipulation alone. Therefore, a snare catheter was introduced via the antegrade system, and the retrograde guidewire was successfully captured and externalized. Subsequently, balloon angioplasty was performed, which resulted in satisfactory revascularization and restoration of antegrade blood flow. A retroflexed ATA represents a unique anatomical challenge that can lead to difficulties in guidewire advancement. In such cases, clear visualization of the vessel route and snare-assisted guidewire externalization is critical determinants of successful revascularization. We must continue to improve the success rate of EVT for infrapopliteal lesions by steadily developing treatment strategies, while carefully examining the advantages and disadvantages of various EVT procedures.

## Introduction

Endovascular treatment (EVT) for infrapopliteal lesions has evolved significantly; however, anatomical variations in the anterior tibial artery (ATA) can complicate revascularization. In particular, a retroflexed ATA origin may hinder proper antegrade guidewire advancement owing to unfavorable angulation; even with a retrograde approach, the guidewire tends to take a subintimal course into the popliteal artery (POPA). Herein, we report a case in which snare-assisted externalization enabled successful EVT in a challenging anatomy.

## Case report

**Fig. 1:**
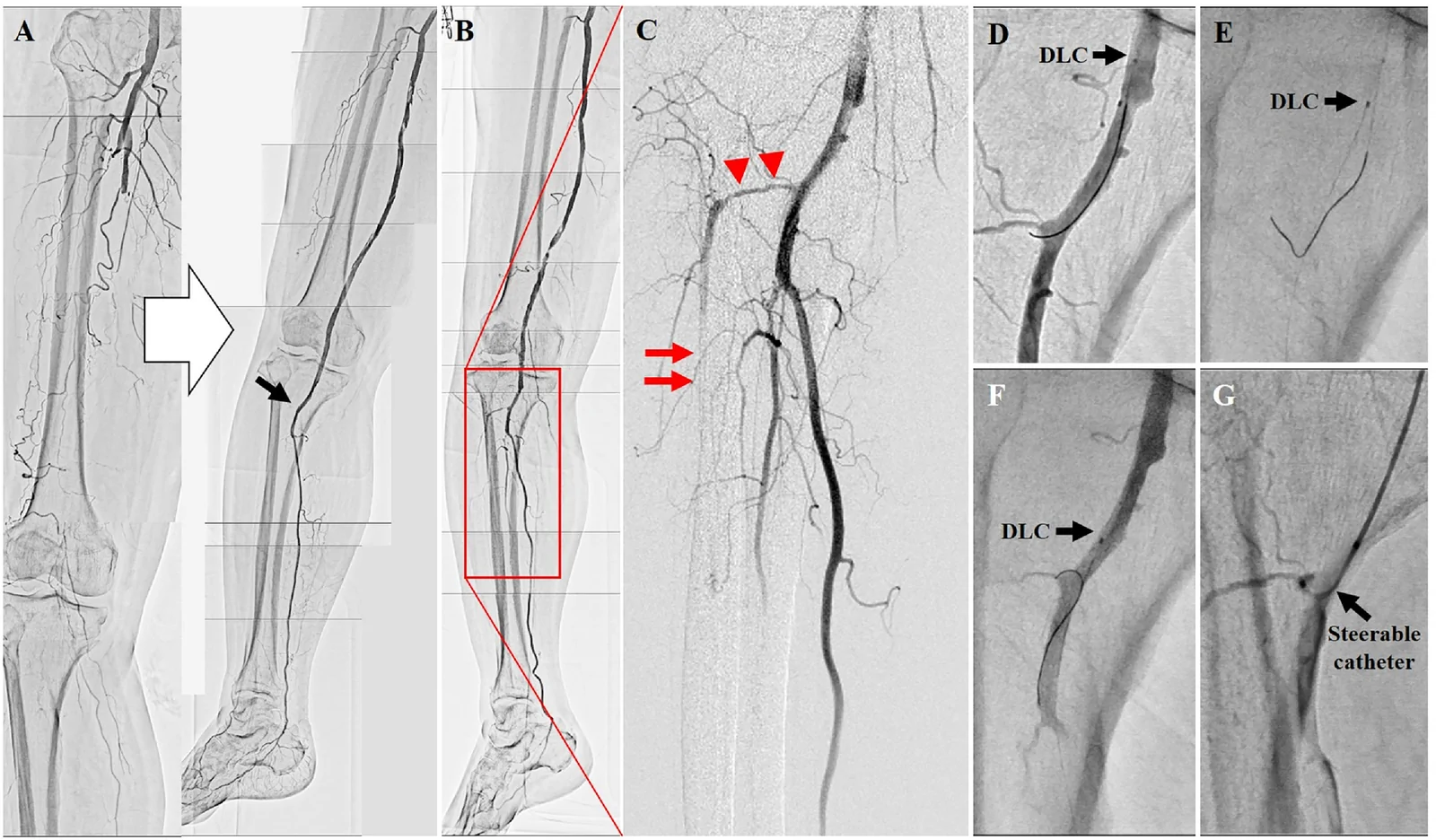
(A) Initial endovascular treatment for the occlusion of the right superficial femoral artery. After procedure, the entry of the anterior tibial artery (ATA) could not be clearly described (arrow). (B) The ATA origin appeared in the angiography in this session. (C) Magnified image of the proximal infrapopliteal artery showing severe stenosis of the ATA (arrowheads). The absence of contrast filling indicates ATA occlusion (arrows). (D) Antegrade guidewire advancement toward the ATA ostium using a dual-lumen microcatheter (DLC). (E) The larger second curve of the guidewire did not function properly. (F) The reverse guidewire technique with DLC was ineffective. (G) The steerable catheter also functions inadequately.

An 86-year-old man presented with pain in the right lower extremity (Rutherford category 4). Initial angiography showed total occlusion at proximal lesion of the superficial femoral artery, and we successfully established one straight line to the posterior tibial artery. The final angiography could not demonstrate ATA just from the ostium (Fig. 1A). This treatment made the ankle-brachial pressure index (ABI) on the right side improved from unmeasurable level to 1.00. However, the patient’s symptom persisted during 2 months after initial EVT, and the skin perfusion pressure remained around 36 mmHg. Therefore, we performed complete revascularization of the right lower extremity expecting to relieve symptoms. The patient was administered 100 mg of aspirin and 75 mg of clopidogrel before catheterization. An ipsilateral common femoral artery access system was established using a 4.5Fr Parent Plus guide sheath (MEDIKIT, Tokyo, Japan). Immediately after guide sheath insertion, an intra-arterial bolus of 5000 units of unfractionated heparin was administered. The activated clotting time was maintained at ≥250 s during the procedure. Angiography revealed severe stenosis at the proximal ATA, and vessel occlusion was suspected due to the absence of contrast filling (Fig. 1A and B, arrows). Initially, we did not recognize the exact branching morphology of the ATA from the POPA. Therefore, we attempted an ipsilateral antegrade approach. However, multiple wiring strategies using different guidewires and techniques failed to determine the origin (Fig. 1C–F).

**Fig. 2:**
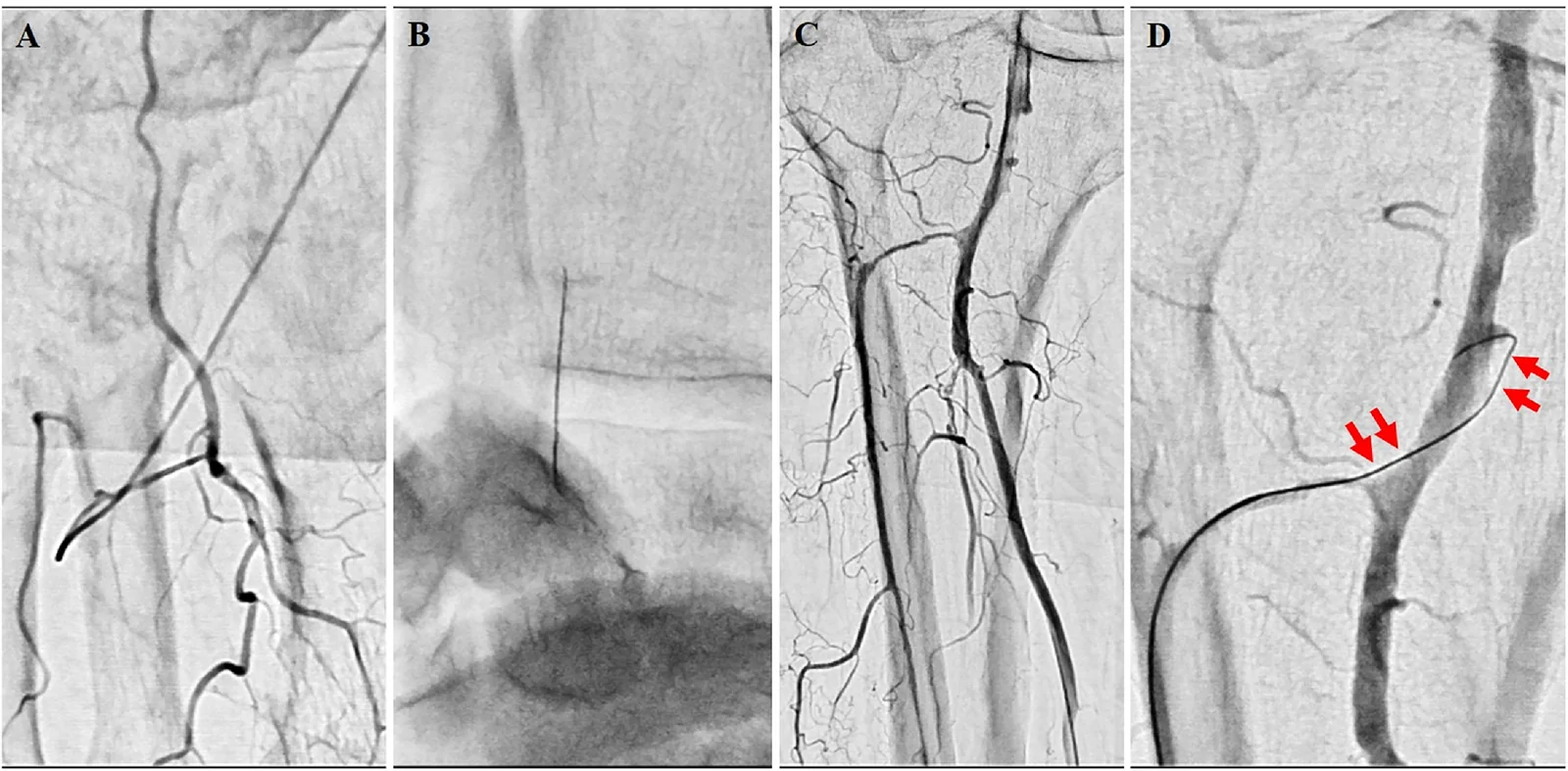
(A) Contrast injection into the pedal arch, visualizing the dorsalis pedis artery (DA). (B) Puncture of the DA under angiography and insertion of the retrograde guidewire. (C) Bidirectional simultaneous angiography demonstrating proximal ATA narrowing. (D) Subintimal advancement of the retrograde guidewire toward the popliteal artery (POPA) (arrows).

Subsequently, a retrograde approach was established. Since the initial angiography demonstrated contrast defect distally to the mid ATA as far as dorsalis pedis artery (DA), a Caravel microcatheter (ASAHI INTECC, Aichi, Japan) was advanced to the pedal arch via the lateral plantar artery to confirm the patency of the DA by injecting contrast medium (Fig. 2A). Then, we punctured the DA under angiography (Fig. 2B), and an Ichibanyari PAD microcatheter (Kaneka Medix, Osaka, Japan) was introduced into the proximal ATA. Simultaneous bidirectional angiography revealed that the ATA was an open vessel with narrowing at the proximal site (Fig. 2C). A Cruise guidewire (ASAHI INTECC) was initially advanced toward the POPA; however, it was apparently outside the vessel lumen (Fig. 2D arrows).

**Fig. 3:**
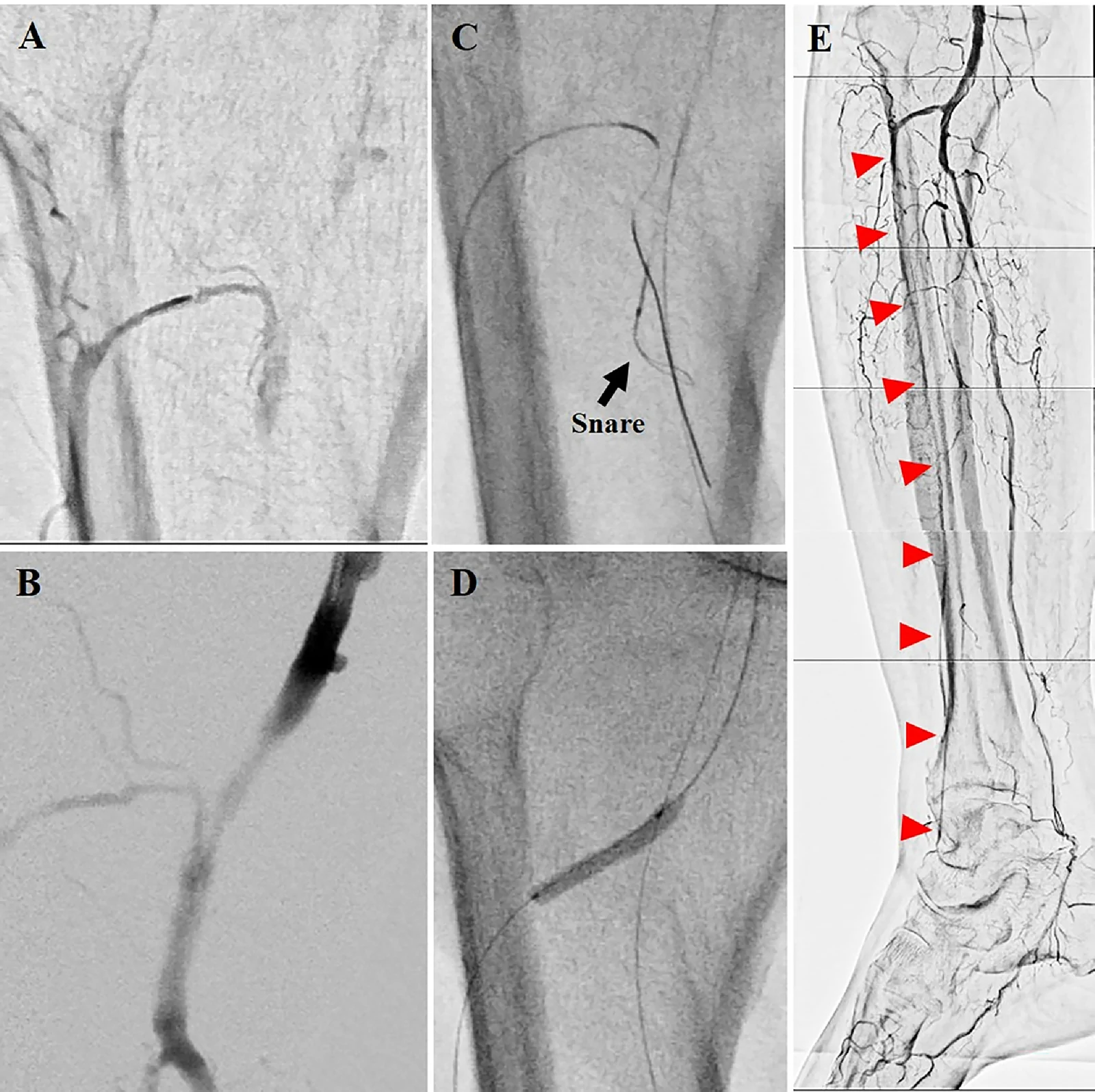
(A) Retrograde angiography demonstrating the retroflexed ATA origin from the POPA. (B) Reassessment of the initial angiography frame-by-frame, showing the retroflexed ATA ostium. (C) Snare wire capturing the intraluminally advanced retrograde guidewire. (D) Balloon angioplasty of the proximal ATA. (E) Final angiography depicting good vascular patency and favorable blood flow in the ATA (arrowheads).

We examined the angiography frame-by-frame and found that the ATA originated from the POPA in a retroflexed configuration (Fig. 3A and B). A Jupiter FC guidewire (Boston Scientific, MA) was advanced intraluminally toward the tibioperoneal trunk; however, reversing its direction proximally was difficult. To overcome this limitation, a snare catheter was introduced using an antegrade approach. The retrograde guidewire was successfully captured and externalized through the antegrade guide sheath (Fig. 3C). After establishing the antegrade guidewire system, a 3.0/20mm NSE PTA balloon catheter (NIPRO, Osaka, Japan) was inflated 3 times for total 360 seconds at maximum 14 atmospheres around the proximal part of the ATA (Fig. 3D). Final angiography demonstrated satisfactory revascularization of the ATA, with less than 30% residual stenosis, no significant dissection, and improvement in antegrade blood flow (Fig. 3E arrowheads). Both the DA and common femoral artery puncture sites were completed hemostasis by manual compression and no entry site complication was observed.

After the treatment, patient’s symptoms disappeared. Dual antiplatelet therapy with 100 mg of aspirin and 75 mg of clopidogrel was conducted for 3 months, following clopidogrel monotherapy thereafter. No symptoms thought to be related to chronic limb-threatening ischemia have appeared since then for more than 4 years.

## Discussion

Here, we report a successful case of EVT in which a rare retroflexed ATA origin with complex anatomy hindered smooth guidewire passage in both the antegrade and retrograde approaches. Although EVT of lower-extremity arterial disease is expected to have higher revascularization success rates, there are several pitfalls in the specific situations of EVT for infrapopliteal lesions [1]. The ATA is usually the first branch of the POPA, and its branching angle tends to be large. Under such circumstances, a retroflexed ATA origin is a rare clinical entity, and there is no report regarding such anatomical variant of the ATA although anatomic variations of popliteal artery were evaluated by computed tomography (CT) angiography on a large scale [2,3]. An antegrade approach to the retroflexed ATA origin, which branches from a relatively large POPA, can sometimes be difficult, making a retrograde approach promising, as in our case. We tried several antegrade wiring techniques, such as the reverse guidewire technique and the LEONIS Mova steerable catheter (SB-KAWASUMI LABORATORIES, Kanagawa, Japan) [4]. The former technique was established in 2008 and is widely used in the field of coronary intervention [5,6]. The latter device has been reported in the field of cerebrovascular diseases; however, both procedures did not work well in this case. Since we initially failed to obtain a clear image of the branching configuration of the ATA using solely antegradely performed angiography (Fig. 1D, F, and G), we wasted time manipulating the ipsilateral antegrade wiring. Precise angiography or intravascular ultrasound should be performed at the origin in the early stages. IVUS is reported to be sometimes useful for identifying the angiographically ambiguous entry of a branching vessel [7]. Moreover, multiple anatomical and diagnostic imaging studies demonstrate that preprocedural assessment of the branching patterns of the popliteal artery using CT angiography is crucial for planning EVT procedure [2,3]. However, we missed the opportunity to check either the IVUS or CT angiography in this case.

Several options are available for retrograde access, including transcollateral, transpedal, and distal punctures. We initially used the transpedal approach, and pedal arch angiography using a microcatheter visualized the relatively healthy DA. We then switched to distal puncturing of the DA because we had predicted the need for sensitive retrograde guidewire handling [8]. Initially, the retrograde guidewire was advanced toward the POPA in a straightforward manner at the bifurcation; however, the guidewire appeared to take a subintimal course into the POPA. Finally, we confirmed the origin of the retroflexed ATA from the POPA by retrogradely injecting contrast medium via microcatheter (Fig. 3A and B). Thereafter, retrograde intraluminal guidewire passage was easily achieved; however, cranial directional control of the guidewire remained limited because of anatomical constraints. Therefore, snare-assisted externalization is an effective bailout strategy to convert difficult wiring situations into stable pull-through systems. The final angiographic results were satisfactory after balloon angioplasty. However, clinical concerns regarding the long-term patency of the ATA ostium remain because retroflexed anatomical features persist.

Herein, we present a case of lower-extremity artery disease with a retroflexed ATA origin, in which clear visualization of the vessel route and snare-assisted guidewire externalization were critical determinants of successful revascularization. We must continue to improve the success rate of EVT for infrapopliteal lesions by steadily developing treatment strategies, while carefully examining the advantages and disadvantages of various EVT procedures.

## Ethical approval

All procedures were performed in accordance with the ethical standards of our institution and the 1964 Declaration of Helsinki. Informed consent was obtained from the patient for the publication of this case report and the accompanying images. A copy of the written consent form is available for review by the journal’s editor.

## Author contributions

TN is the primary author of this study. SK, TB, TM, MI, JM, HK, KN, and TT substantially revised the manuscript. NW contributed significantly to the conception and design of this study. All the authors have read and approved the final version of the manuscript.

## Patient consent

Written informed consent for the publication of this case report was obtained from the patient.
